# Correction to Energy transfer between Tb^3+^ and Eu^3+^ in LaPO_4_: Pulsed Versus Switched‐Off Continuous Wave Excitation

**DOI:** 10.1002/advs.202404474

**Published:** 2024-05-13

**Authors:** Y. Luo, Z. Liu, H.‐T. Wong, L. Zhou, K.‐L. Wong, K. K. Shiu, P. A. Tanner

Energy transfer between Tb^3+^ and Eu^3+^ in LaPO_4_: pulsed versus switched‐off continuous wave excitation. *Advanced Science* 1900487 (1‐12), 2019. *Advanced Science* **2019**; *6*: 1900487 (1‐12).

There is a typographic error in Equation (5), which should read as “*τ*_D_(*s*‐3)*^k^* ^+1^ *R^s^* ^‐3^” and not as written “*τ*_D_^(^ *^s^* ^‐3)^ *^k^* ^+1^ *R^s^* ^‐3^”. Also, the headings in Table 1 are “[ms]^−1^” and not “[ms^−1^]”.

We apologize for this error.

